# ON THE POSSIBILITY TO RESOLVE GADOLINIUM- AND CERIUM-BASED CONTRAST AGENTS FROM THEIR CT NUMBERS IN DUAL-ENERGY COMPUTED TOMOGRAPHY

**DOI:** 10.1093/rpd/ncab078

**Published:** 2021-06-09

**Authors:** Alexandr Malusek, Lilian Henriksson, Peter Eriksson, Nils Dahlström, Åsa Carlsson Tedgren, Kajsa Uvdal

**Affiliations:** Department of Health, Medicine and Caring Sciences, Linköping University, SE-581 83, Linköping, Sweden; Center for Medical Image Science and Visualization (CMIV), Linköping University, SE-581 83, Linköping, Sweden; Center for Medical Image Science and Visualization (CMIV), Linköping University, SE-581 83, Linköping, Sweden; Department of Physics, Chemistry and Biology, Linköping University, SE-581 83, Linköping, Sweden; Department of Health, Medicine and Caring Sciences, Linköping University, SE-581 83, Linköping, Sweden; Center for Medical Image Science and Visualization (CMIV), Linköping University, SE-581 83, Linköping, Sweden; Department of Health, Medicine and Caring Sciences, Linköping University, SE-581 83, Linköping, Sweden; Center for Medical Image Science and Visualization (CMIV), Linköping University, SE-581 83, Linköping, Sweden; Department of Medical Radiation Physics and Nuclear Medicine, Karolinska University Hospital, SE-171 77 Stockholm, Sweden; Department of Physics, Chemistry and Biology, Linköping University, SE-581 83, Linköping, Sweden

## Abstract

Cerium oxide nanoparticles with integrated gadolinium have been proved to be useful as contrast agents in magnetic resonance imaging. Of question is their performance in dual-energy computed tomography. The aims of this work are to determine (1) the relation between the computed tomography number and the concentration of the I, Gd or Ce contrast agent and (2) under what conditions it is possible to resolve the type of contrast agent. Hounsfield values of iodoacetic acid, gadolinium acetate and cerium acetate dissolved in water at molar concentrations of 10, 50 and 100 mM were measured in a water phantom using the Siemens SOMATOM Definition Force scanner; gadolinium- and cerium acetate were used as substitutes for the gadolinium-integrated cerium oxide nanoparticles. The relation between the molar concentration of the I, Gd or Ce contrast agent and the Hounsfield value was linear. Concentrations had to be sufficiently high to resolve the contrast agents.

## INTRODUCTION

Contrast agents are used in computed tomography (CT) to enhance the visibility of internal structures of the patient body. At present, the most often used contrast agents are iodine or barium-based; see refs^(1,2)^ for information on other agents. Iodine-based (*Z*_I_ = 53) contrast agents are often used for intra-arterial and intravenous examinations. Barium-based (*Z*_Ba_ = 56) contrast agents are taken orally as a suspension for the imaging of the gastrointestinal tract; their use in CT examinations is less frequent nowadays, nevertheless they may be present from previous planar radiography examinations. An application of a contrast agent is associated with a risk of adverse reactions^(3)^, for instance the use of an iodinated contrast agent may lead to an impairment in renal function^(4)^. Though many of the adverse reactions can be treated, there is a risk of for instance permanent renal damage. For this reason, there is an interest in developing safer contrast agents.

In the case of a single-energy CT (SECT) examination, the patient is typically scanned before and after the injection of the full dose of the contrast agent into the blood vessel^(5)^. In the case of a dual-energy CT (DECT)^(6)^ examination, the scanning can be done after the injection of the contrast agent; a material decomposition can be used to calculate the concentration of iodine and the image without the contrast agent (virtual non-contrast image) can be computed by the CT scanner^(7)^. By eliminating the scan before the application of the contrast agent, this approach saves both time in the scanner and patient dose. In the case of the emerging photon counting CT with energy resolving detectors^(8)^, several different contrast agents can be applied and tracked simultaneously. Clinical benefits of this approach are being studied.

Currently used clinical iodinated agents suffer from several limitations: (1) they are rapidly excreted by the kidney due to their low molecular weight, (2) large doses are required and may lead to adverse effects and (3) targeted imaging is difficult to achieve. Contrast agents based on nanoparticles may overcome these problems^(9)^. Moreover, nanoparticle-based contrast agents may be used for K-edge imaging in spectral CT.

Development of clinically applicable contrast agents is a demanding process. The solutions should be chemically stable, nontoxic to patients and isotonic. Salts that are added to achieve an isotonic solution complicate theoretical calculations of attenuation properties of the contrast agent as their amounts are often not specified by the manufacturers. In this work, we wanted to evaluate the attenuation properties of Gd and Ce based contrast agents in relation to the iodine-based ones. The authors have developed nanoparticle-based contrast agents using these two elements for magnetic resonance imaging (MRI)^(10)^. Of interest was the performance of such contrast agents in CT.

## THEORY

The Hounsfield value, $H$, is defined as

(1) \begin{equation*} H=1000\left(\mu /{\mu}_w-1\right), \end{equation*}

where $\mu$ and ${\mu}_w$ are the linear attenuation coefficients of the imaged material and water, respectively. For water and air, the Hounsfield values are 0 and −1000 HU, respectively. The Hounsfield value is well defined for monoenergetic photon beams. For polyenergetic beams, which are used in medical CT scanners, the Hounsfield value is averaged over the energy spectrum of photons; it depends on the filtration of the photon beam both by the X-ray tube filters and the imaged object^(11)^. To a certain degree, it also depends on the reconstruction algorithm^(12)^.

In the independent atom approximation, the linear attenuation coefficient of a compound or mixture can be calculated as^(13)^

(2) \begin{equation*} \frac{\mu }{\rho }={\sum}_i{w}_i\frac{\mu_i}{\rho_i} \end{equation*}

where $\rho$ is the mass density of the voxel material, and ${w}_i$ and ${\mu}_i/{\rho}_i$ are the mass fraction and mass attenuation coefficient, respectively, of the *i*th constituent (typically an element or base material) of the mixture. By dividing Equation (2) with the linear attenuation coefficient of water, ${\mu}_w$, we get after some algebraic manipulations

(3) \begin{eqnarray*} H\!\!\!\!\!\!&=&\!\!\!\!\!\!{\sum}_i{w}_i\frac{\rho }{\rho_i}{H}_i+1000\left({\sum}_i{w}_i\frac{\rho }{\rho_i}-1\right)\nonumber\\\!\!\!\!\!\!&=&\!\!\!\!\!\!{\sum}_i{v}_i{H}_i+1000\left({\sum}_i{v}_i-1\right), \end{eqnarray*}

where $H$ and ${H}_i$ are the Hounsfield values of the voxel and the *i*th constituent, respectively, and ${v}_i$ is the partial volume of *i*th constituent. If partial volumes of individual constituents are preserved in the mixture, then ${\sum}_i{v}_i=1$ and the last term in Equation (3) disappears. In practical situations, however, the sum of partial volumes may not equal one. For an iodinated contrast agent dissolved in water, Equation (3) can be written as

(4) \begin{equation*} H={v}_{\mathrm{w}}{H}_{\mathrm{w}}+{v}_{\mathrm{I}}{H}_{\mathrm{I}}+\Delta H={v}_{\mathrm{I}}{H}_{\mathrm{I}}+\Delta H, \end{equation*}

where the indices w and I stand for water and iodine, respectively, and $\Delta H$ represents the last term in Equation (3). We recall that ${H}_{\mathrm{w}}=0$ HU. In practice, the amount of the contrast agent is typically measured in M = mol/l. The volume fraction can be written as

(5) \begin{equation*} {v}_i=\frac{V_i}{V}=\frac{V_i}{n_{\mathrm{mol},i}}\frac{n_{\mathrm{mol},i}}{V}={V}_{\mathrm{mol},i}\cdot{c}_i, \end{equation*}

where $V$, ${V}_i$ and ${V}_{\mathrm{mol},i}$ are the total, partial and molar volumes, respectively, ${n}_{\mathrm{mol},i}$ is the amount of the *i*th constituent in mol, and ${c}_i={n}_{\mathrm{mol},i}/V$ is the concentration of substance in M. By inserting Equation (5) to Equation (4) we get

(6) \begin{equation*} H={V}_{\mathrm{mol},\mathrm{I}}\cdot{H}_{\mathrm{I}}\cdot{c}_{\mathrm{I}}+\Delta H={a}_{\mathrm{I}}\cdot{c}_{\mathrm{I}}+\Delta H, \end{equation*}

where the factor ${a}_I={V}_{\mathrm{mol},\mathrm{I}}\cdot{H}_{\mathrm{I}}$ (typically specified in HU/mM) does not depend on the concentration of iodine. If partial volumes are preserved in the solution, then $\Delta H=0$ and Equation (6) becomes

(7) \begin{equation*} H={a}_{\mathrm{I}}\cdot{c}_{\mathrm{I}}, \end{equation*}

i.e. the Hounsfield value is proportional to the molar concentration of the iodine-based contrast agent.

Obviously, the correction term $\Delta H$ in Equation (6) equals 0 when there is no iodine in the solution. Since $\Delta H=\Delta H({c}_{\mathrm{I}})$ should be a continuous function of ${c}_{\mathrm{I}}$, $\Delta H({c}_{\mathrm{I}})$ approaches 0 as ${c}_{\mathrm{I}}$ approaches zero. Also, note that ${c}_{\mathrm{I}}$ will reach its maximum when the solution consists of the iodine-based contrast agent only. In clinically relevant situations, however, ${c}_{\mathrm{I}}$ is much lower than the maximum concentration, and the linear relation in Equation (7) can be expected.

In Equation (7), the concentration of iodine ${c}_{\mathrm{I}}$ is given in M, i.e. mol/l. An alternative quantity used by some authors is the partial density of iodine ${\rho}_i$ given in mg/ml. The relation between these two quantities is

(8) \begin{equation*} {\rho}_I=\frac{n_{\mathrm{mol},\mathrm{I}}\cdot{m}_{\mathrm{mol},\mathrm{I}}}{V}={c}_I\cdot{m}_{\mathrm{mol},\mathrm{I}}, \end{equation*}

where ${m}_{\mathrm{mol},\mathrm{I}}$ is the molar weight (in mol/g) of iodine; ${n}_{\mathrm{mol},\mathrm{I}}$ and $V$ have the same meaning as in Equation (5). Equation (7) then becomes

(9) \begin{equation*} H={a}_{\mathrm{I}}\cdot{c}_{\mathrm{I}}=\frac{a_{\mathrm{I}}}{m_{\mathrm{mol},\mathrm{I}}}\cdot{c}_{\mathrm{I}}{m}_{\mathrm{mol},\mathrm{I}}={a}_{\rho, I}\cdot{\rho}_I, \end{equation*}

where ${a}_{\rho, I}={a}_{\mathrm{I}}/{m}_{\mathrm{mol},\mathrm{I}}$ is the coefficient of proportionality in HU/(mg/ml).

## MATERIALS AND METHODS

To overcome the problem with inaccurately known amounts of additional substances in commercially available contrast agents, chemical compounds with well-known elemental compositions (Table 1) were used for both theoretical predictions and practical measurements of the attenuation properties. Both cerium acetate and gadolinium acetate are hygroscopic. In this work, it was assumed that dry substances were used.

**Table 1:** Contrast agent substitutes. Molecular formulas and molar masses, ${m}_{\mathrm{mol}}$, also known as molecular weights were taken from the PubChem database.

	Molecular formula	${m}_{\mathrm{mol}}$ (g/mol)
Iodoacetic acid	C_2_H_3_IO_2_	185.95
Cerium acetate	Ce(CH_3_CO_2_)_3_	317.25
Gadolinium acetate	Gd(CH_3_CO_2_)_3_	334.38

### Computed Hounsfield values

The concentration of the contrast agent was specified as the molarity (in mol/l) of the contrast agent element. For the iodoacetic acid (IA), the mass fraction, ${w}_{\mathrm{IA}}$, of the molecule in the solution was then calculated as

(8) \begin{equation*} {w}_{\mathrm{solution}}\left(\mathrm{IA}\right)=\frac{m\left(\mathrm{IA}\right)}{m}=\frac{m\left(\mathrm{IA}\right)}{\rho_{\mathrm{w}}{V}_{\mathrm{w}}+m\left(\mathrm{IA}\right)}, \end{equation*}

where $m(\mathrm{IA})$ is the mass of the contrast agent molecules in the solution, $m$ is the mass of the solution, ${\rho}_{\mathrm{w}}$ is the mass density of water and ${V}_{\mathrm{w}}$ is the volume of water in the solution. Accurate estimation of the volume of water ${V}_{\mathrm{w}}$ is difficult, more information is in the Discussion section. Since the concentration of the contrast agent was small, ${V}_{\mathrm{w}}$ was approximated by the volume of the solution $V$. Mass of the contrast agent molecules was calculated as

(9) \begin{eqnarray*} m\left(\mathrm{I}\mathrm{A}\right)\!\!\!\!\!\!&=&\!\!\!\!\!\!\frac{m\left(\mathrm{I}\right)}{w_{\mathrm{IA}}\left(\mathrm{I}\right)}=\frac{n\left(\mathrm{I}\right)\cdot{m}_{\mathrm{mol}}\left(\mathrm{I}\right)}{w_{\mathrm{IA}}\left(\mathrm{I}\right)}\nonumber\\\!\!\!\!\!\!&=&\!\!\!\!\!\!\frac{c_I\cdot V\cdot{m}_{\mathrm{mol}}\left(\mathrm{I}\right)}{w_{\mathrm{IA}}\left(\mathrm{I}\right)}, \end{eqnarray*}

where $m(\mathrm{I})$ is the mass of all contrast agent atoms in the solution, ${w}_{\mathrm{IA}}(\mathrm{I})$ is the mass fraction of the contrast agent atom in the contrast agent molecule, $n(\mathrm{I})$ is the amount of the contrast agent in mol, and ${m}_{\mathrm{mol}}(\mathrm{I})$ is the mass of 1 mol of the contrast agent atoms. Note that the volume $V$ of the solution cancels out when Equation (9) is inserted to Equation (8) and the assumption ${V}_{\mathrm{w}}=V$ is made. Mass fractions, ${w}_i$, of individual atoms in the molecule of the contrast agent were calculated as

(10) \begin{eqnarray*} {w}_{\mathrm{IA}}\left({X}_i\right)&=&\frac{a_{\mathrm{IA}}\left({X}_i\right)m\left({X}_i\right)}{\sum_j{a}_{\mathrm{IA}}\left({X}_j\right)m\left({X}_j\right)}\nonumber\\&=&\frac{a_{\mathrm{IA}}\left({X}_i\right){A}_{\mathrm{r}}\left({X}_i\right)}{\sum_j{a}_{\mathrm{IA}}\left({X}_j\right){A}_{\mathrm{r}}\left({X}_j\right)}, \end{eqnarray*}

where ${a}_{\mathrm{IA}}({X}_i)$ is the number of atoms of the element ${X}_i$ (e.g. H, C, O, …) in the molecule, $m({X}_i)$ is the mass of the atom and ${A}_{\mathrm{r}}({X}_i)$ is the relative atomic mass of the atom.

Elemental mass fraction, ${w}_{\mathrm{solution}}({X}_i)$, of atom ${X}_i$ in the solution was calculated as

(11) \begin{eqnarray*} {w}_{\mathrm{solution}}\left({X}_i\right)\!\!\!\!\!\!&=&\!\!\!\!\!\!{w}_{\mathrm{solution}}\left(\mathrm{IA}\right){w}_{\mathrm{IA}}\left({X}_i\right)\\\nonumber&&\!\!\!\!\!\!+{w}_{\mathrm{solution}}\left(\mathrm{water}\right){w}_{\mathrm{water}}\left({X}_i\right) \end{eqnarray*}

where ${w}_{\mathrm{IA}}({X}_i)$ is the mass fraction of element ${X}_i$ in the contrast agent molecule (Equation (8)), ${w}_{\mathrm{solution}}(\mathrm{water})=1-{w}_{\mathrm{solution}}(\mathrm{IA})$ is the mass fraction of water in the solution and ${w}_{\mathrm{water}}({X}_i)$ is the mass fraction of element ${X}_i$ in the water molecule. Elemental mass attenuation coefficients were taken from the XCOM database (https://www.nist.gov/pml/xcom-photon-cross-sections-database).

The linear attenuation coefficient of the solution was then calculated as

(12) \begin{equation*} \mu ={\rho}_{\mathrm{solution}}\frac{\mu }{\rho }, \end{equation*}

where the mass attenuation coefficient $\mu /\rho$ was calculated from Equation (2). The density of the solution was estimated as

(13) \begin{equation*} {\rho}_{\mathrm{solution}}=\frac{\rho_{\mathrm{w}}{V}_{\mathrm{w}}+m\left(\mathrm{IA}\right)}{V}, \end{equation*}

where the assumption ${V}_{\mathrm{w}}=V$ was made (see Equation (8)). Calculations for cerium acetate and gadolinium acetate were performed similarly.

### Measured Hounsfield values

Hounsfield values of IA, gadolinium acetate and cerium acetate dissolved in MilliQ-water at molar concentrations of 10, 50 and 100 mM were measured in a cylindrical water phantom with the diameter of 20 cm using the Siemens SOMATOM Force scanner. Partial densities corresponding to these molar concentrations are listed in Table 2; they were derived using the molar masses of 126.90, 140.12 and 157.20 g/mol for I, Ce and Gd, respectively. For each solution, one vial (diameter of 16 mm) containing the contrast agent solution was placed in the center of the cylindrical water phantom; the axis of the phantom was slightly tilted to move air bubbles to the end of the vial.

**Table 2:** Molar concentrations ${c}_i$ and corresponding partial densities ${\rho}_i$ for I, Ce and Gd.

${c}_i$ (mM)	${\rho}_{\mathrm{I}}$ (mg/ml)	${\rho}_{\mathrm{Ce}}$ (mg/ml)	${\rho}_{\mathrm{Gd}}$ (mg/ml)
10	1.27	1.40	1.57
50	6.35	7.01	7.86
100	12.69	14.01	15.72

**Figure 1:**
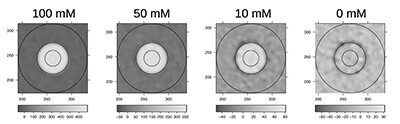
Examples of circles defining inner regions for the Ce contrast agent with concentrations of 100, 50, 10 and 0 mM.

For 100-mM solutions, SECT scans with tube voltages of 70, 80, 90, 100, 110, 120, 130, 140 and 150 kV were taken. For all solutions (10, 50 and 100 mM), DECT scans using 80 kV and Sn150kV were taken. Images were reconstructed using the ADMIRE algorithm with strength 5 and the Qr32s kernel. Voxels inside the inner circular region in Figure 1 were selected for further processing; diameter of the circle was smaller than the diameter of the vial to avoid effect close to its walls. To compensate for the tilt of the phantom axis, the center of the circle was obtained by interpolation between the first and last processed slice. Typically, 80 slices with the thickness of 1 mm were processed for each vial (~50 000 voxels).

## RESULTS

**Figure 2:**
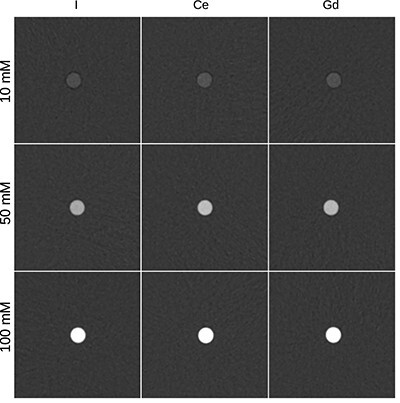
Reconstructed images for I, Ce and Gd (left, middle and right-side column) with concentrations of 10, 50 and 100 mM (top, middle and bottom row) for the tube voltage of 80 kV. The image size was 120 mm × 120 mm. The window width of 600 HU and centre of 170 HU corresponded to a typical angiography examination.

Reconstructed images for I, Ce and Gd with concentrations of 10, 50 and 100 mM and the tube voltage of 80 kV are plotted in Figure 2.

**Figure 3:**
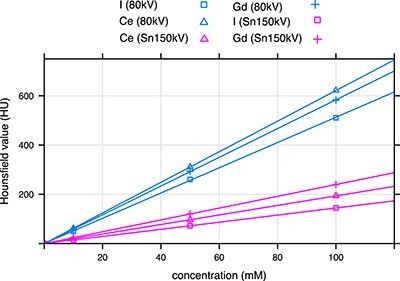
Hounsfield value as a function of the concentration of the I, Ce and Gd contrast agent for the tube voltages of 80 kV and Sn150 kV.

Hounsfield values as functions of the concentration of the contrast agent for I, Ce and Gd and the tube voltages of 80 kV and Sn150kV are shown in Figure 3. Measured points were fitted with linear models using the lm() function in R (https://www.r-project.org/). The fits show that the linearity predicted by Equation (7) was fulfilled. Note that the measured average Hounsfield values were associated with low uncertainties since the sample contained large number of voxels. These uncertainties were much lower than the ones associated with reproducibility of the CT scanner measurements.

**Figure 4:**
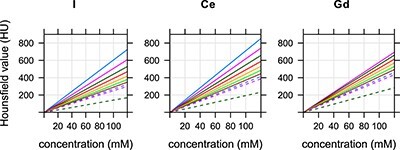
Hounsfield value as a function of concentration for all tube voltages and the I, Ce and Gd solutions. Curves from top to bottom correspond to 70, …, 150 kV. For Gd, the curve for 80 kV overlaps the one for 70 kV.

Hounsfield values as functions of concentration for the I, Ce and Gd contrast agents and tube voltages of 70, …, 150 kV are shown in Figure 4. These curves were obtained in R using the linear model in Equation (7) and measured data for the concentration of 100 mM. Corresponding coefficients of proportionality are listed in Table 3.

**Table 3:** Coefficients of proportionality $\mathrm{a}$ in the $H=\mathrm{ac}$ relation for I, Ce and Gd. Associated standard uncertainties are denoted $\mathrm{u}(\mathrm{a})$. All values are in HU/mM.

kVp (kV)	${a}_{\mathrm{I}}$	$u({a}_{\mathrm{I}})$	${a}_{\mathrm{Ce}}$	$u({a}_{\mathrm{Ce}})$	${a}_{\mathrm{Gd}}$	$u({a}_{\mathrm{Gd}})$
70	6.0431	0.0023	7.0973	0.0026	5.7966	0.0090
80	5.0738	0.0019	6.1780	0.0057	5.7912	0.0048
90	4.4073	0.0004	5.4748	0.0043	5.5089	0.0116
100	3.9109	0.0057	4.9011	0.0095	5.1258	0.0083
110	3.4891	0.0032	4.4278	0.0054	4.7573	0.0011
120	3.1602	0.0002	4.0433	0.0100	4.4157	0.0017
130	2.9017	0.0046	3.7156	0.0005	4.1242	0.0017
140	2.6905	0.0031	3.4482	0.0065	3.8431	0.0001
150	2.4960	0.0095	3.2228	0.0000	3.6223	0.0007
Sn150	1.4076	0.0025	1.9350	0.0027	2.3663	0.0039

**Figure 5:**
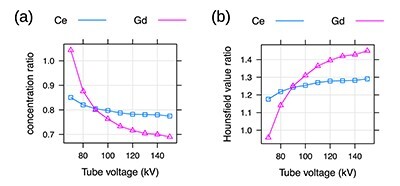
(**a**) The ${\mathrm{c}}_{\mathrm{X}}/{\mathrm{c}}_{\mathrm{I}}$ ratio, where $\mathrm{X}$ stands for Ce or Gd, resulting in the same Hounsfield value 𝐻. (**b**) The ${\mathrm{H}}_{\mathrm{X}}/{\mathrm{H}}_{\mathrm{I}}$ ratio for the same molar concentration $\mathrm{c}$.

Concentrations of Ce and Gd relative to the concentration of I that produce the same Hounsfield value are shown in Figure 5(a). Figure 5(b) shows the Hounsfield values of Ce and Gd relative to that of I for solutions with the same concentrations.

**Figure 6:**
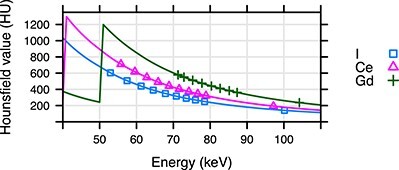
Hounsfield value as a function of photon energy for 100-mM solution of I, Ce and Gd. Measured values are plotted as markers, computed values derived from tabulated data are plotted as solid lines. K-edge energies for _53_I, _58_Ce and _64_Gd are 33.2, 40.5 and 50.2 keV, respectively.

Table 4 shows average energies of X-ray tube energy spectra and corresponding contrast agent specific effective energies, ${E}_{\mathrm{eff}}$, of the CT scanner for the I, Ce and Gd solutions; the effective energy was defined as the energy for which the measured Hounsfield value equaled the tabulated value, ${H}_{\mathrm{measured}}={H}_{\mathrm{tabulated}}\ ({E}_{\mathrm{eff}})$. Figure 6 illustrates differences in Hounsfield values arising from the differences in effective energies.

**Table 4:** Average energies, ${E}_{\mathrm{avg}}$, of X-ray tube energy spectra and corresponding contrast agent specific effective energies, ${E}_{\mathrm{eff}}$, of the CT scanner for the I, Ce and Gd solutions.

kVp (kV)	${E}_{\mathrm{avg}}$ (keV)	${E}_{\mathrm{eff},\mathrm{I}}$ (keV)	${E}_{\mathrm{eff},\mathrm{Ce}}$ (keV)	${E}_{\mathrm{eff},\mathrm{Gd}}$ (keV)
70	47.96	52.9	55.8	71.3
80	52.08	57.4	59.6	71.3
90	55.65	61.3	62.8	72.9
100	58.73	64.5	65.9	75.2
110	61.60	67.8	68.8	77.8
120	64.25	70.8	71.6	80.3
130	66.99	73.5	74.2	82.7
140	69.48	76.0	76.6	85.2
150	72.05	78.5	78.9	87.3
Sn150	98.61	100.2	97.2	104.2

**Figure 7:**
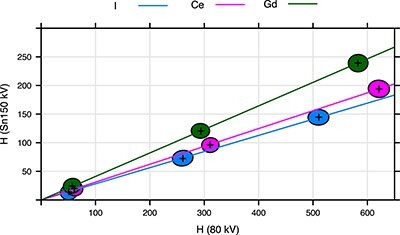
A scatter plot of Hounsfield values at Sn150 kV against Hounsfield values at 80 kV. Instead of individual points for each voxel, covariance ellipses defining 95% confidence regions are plotted for concentrations of 10, 50 and 100 mM. The lines correspond to average values for different molar concentrations of the contrast agent.

The level of noise in the scans using the water cylinder is shown in Figure 7 for the I, Ce and Gd contrast agents scanned at 80 kV and Sn140kV.

## DISCUSSION

The presented data can be used as follows. To achieve the Hounsfield value of 400 HU at 120 kV, the concentration of ~90 ($=400/4.4157$) mM of Gd can be used. A lowering of the tube voltage to 90 kV will increase the Hounsfield value to 495 ($=5.5089\cdot 90$) HU, see Figure 4 and Table 3. To achieve similar Hounsfield values with Gd as with I at 120 kV, the concentration of Gd has to be 0.72 times smaller than the concentration of I, see Figure 5(a).

A derivation similar to the one for a contrast agent in water can be used for a contrast agent in a non-water material. If the Hounsfield value of the material, ${H}_T,$ is not much different from that of water, then ${H}_I-{H}_T\cong{H}_I$ and Equation (4) can be written as $H=(1-{v}_{\mathrm{I}}){H}_{\mathrm{T}}+{v}_{\mathrm{I}}{H}_{\mathrm{I}}={H}_{\mathrm{T}}+{v}_{\mathrm{I}}({H}_I-{H}_T)\cong{H}_{\mathrm{T}}+{a}_{\mathrm{I}}\cdot{c}_{\mathrm{I}}$. In other words, the contrast enhancement in a tissue can be described via the coefficient of proportionality ${a}_{\mathrm{I}}$; the only difference is that the intercept given by ${H}_{\mathrm{T}}$ is non-zero. This fact allows the comparison of ${a}_{\mathrm{I}}$ presented in this work to the coefficients of proportionality presented in literature.

In a phantom study with an iodine contrast agent, Takanami *et al.*^(14)^ showed that the coefficient of proportionality ${a}_{\mathrm{I}}$ is little affected by the position of the contrast agent inside the phantom. Thus, in practice, vials with contrast agents of several different concentrations can be positioned beside the patient during the scan and used for an experimental determination of the coefficients of proportionality $a$. The study used an Omnipaque 300 iodine solution mixed with saline and Siemens SOMATOM Sensation 16 scanner. Despite the differences in experimental setup, their results, for instance, ${a}_{\mathrm{I}}=27.57$ HU/(mg/ml) for the tube voltage of 120 kV agreed well with the value of 24.90 HU/(mg/ml) presented in this work.

Coefficients of proportionality for Gd were measured by Honkanen *et al*.^(15)^. Their value of 43.8 HU/(mg/ml) for Gadoteridol at 70 kV agrees well with the value of 36.9 HU/(mg/ml) presented in this work. Their work demonstrates the use of a dual-contrast technique, where different diffusion rates of the I and Gd contrast agents are used for diagnostic purposes.

Of interest is whether Hounsfield values can be calculated from tabulated values of linear attenuation coefficients. Table 4 shows that air kerma weighted average energies of the X-ray spectra provided by the manufacturer differed from effective energies. The effective energies were contrast agent specific, and thus it was not possible to use one effective photon energy to predict Hounsfield values of different materials even when we considered the same X-ray spectrum and the same size of the phantom. This issue is illustrated in Figure 6. To predict the Hounsfield value accurately, a computer simulation of the projection acquisition and subsequent image reconstruction process is needed.

Each Hounsfield value is affected by noise, which reduces the possibility to determine the concentration of the Gd, Ce and I contrast agents when they are used simultaneously. The noise depends, among other things, on the tube current, the number of analyzed voxels and the anatomy of the surrounding region. This complicates the determination of decision thresholds and detection limits for the contrast agents in general. Figure 7 shows that, in general, it may be difficult to determine whether the voxel contains Gd or Ce for Hounsfield values lower than 500 HU at 80 kV. The image noise corresponded to almost the maximal tube current of the scanner. With similarly attenuating patients, the lower tube current will lead to higher noise. Clearly, the determination of concentration of I, Ce and Gd would be very unreliable for the concentration of 10 mM. For 50 mM, the concentration of I and Ce may be relatively reliable if only those two contrast agents are used. Even better results could be achieved for the concentrations of 100 mM, but even at these concentrations Ce and Gd cannot be reliably resolved. The authors work on a method to quantitively determine the minimal detectable concentrations of simultaneously used contrast agents in specific clinical applications. The method used for one contrast agent^(16,17)^ is not applicable in this case.

## CONCLUSIONS

The relation between the molar concentration of the I, Gd or Ce contrast agent and the CT number was linear. Average energies of the X-ray spectra did not predict Hounsfield values accurately and effective energies were contrast agent specific. Molar concentrations had to be sufficiently high to resolve the contrast agents.
